# High prevalence of unusual *KRAS*, *NRAS*, and *BRAF* mutations in *POLE* *‐* hypermutated colorectal cancers

**DOI:** 10.1002/1878-0261.13257

**Published:** 2022-07-14

**Authors:** Loetitia Favre, Justine Cohen, Julien Calderaro, Adrien Pécriaux, Cong‐Trung Nguyen, Rémi Bourgoin, Laura Larnaudie, Aurélie Dupuy, Marie Ollier, Emmanuèle Lechapt, Ivan Sloma, Christophe Tournigand, Benoit Rousseau, Anaïs Pujals

**Affiliations:** ^1^ Département de Pathologie AP‐HP, Centre Hospitalier Universitaire Henri Mondor Créteil France; ^2^ INSERM, IMRB Univ Paris Est Creteil France; ^3^ Département d'Hématologie Biologique AP‐HP, Centre Hospitalier Universitaire Henri Mondor Créteil France; ^4^ Service d'Oncologie Médicale AP‐HP, Centre Hospitalier Universitaire Henri Mondor Créteil France; ^5^ Mortimer B. Zuckerman Research Center Memorial Sloan Kettering Cancer Center New York NY USA

**Keywords:** colorectal cancers, immunotherapy, POLE, polymerase epsilon

## Abstract

Exonucleasic domain *POLE* (ed*POLE*) mutations, which are responsible for a hypermutated tumor phenotype, occur in 1–2% of colorectal cancer (CRC) cases. These alterations represent an emerging biomarker for response to immune checkpoint blockade. This study aimed to assess the molecular characteristics of ed*POLE*‐mutated tumors to facilitate patient screening. Based on opensource data analysis, we compared the prevalence of ed*POLE* mutations in a control group of unselected CRC patients (*n* = 222) vs a group enriched for unusual *BRAF*/*RAS* mutations (*n* = 198). Tumor mutational burden (TMB) and immune infiltrate of tumors harboring ed*POLE* mutations were then analyzed. In total, 420 CRC patients were analyzed: 11 ed*POLE*‐mutated tumors were identified, most frequently in microsatellite (MMR)‐proficient young (< 70 years) male patients, with left‐sided tumors harboring noncodon 12 *KRAS* mutation. The prevalence of ed*POLE*‐mutated tumors in the control vs the experimental screening group was, respectively, 0.45% (*n* = 1) vs 5.0% (*n* = 10). Among the 11 ed*POLE*‐mutated cases, two had a low TMB, three were hypermutated, and six were ultramutated. Ed*POLE*‐mutated cases had a high CD8^+^ tumor‐infiltrating lymphocyte (TIL) infiltration. These clinicopathological and molecular criteria may help to identify ed*POLE* mutations associated with a high TMB in CRC, and improve the selection of patients who could benefit from immunotherapy.

## Graphical abstract

Exonucleasic domain *POLE* (edPOLE) mutations, which occur in 1–2% of colorectal cancer, represent an emerging biomarker for response to immunotherapy. This study aimed to assess the molecular characteristics of ed*POLE*‐mutated tumors. Results show that the prevalence of ed*POLE*‐mutated tumors is higher in microsatellite‐proficient young Male patients harboring non‐codon 12 *KRAS* mutations. Ed*POLE*‐mutated cases had a high CD8^+^ lymphocyte infiltration.

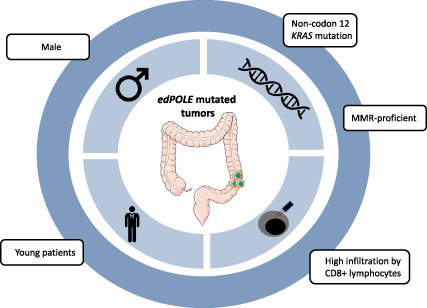

**Abbreviations**
CRC: colorectal cancer
edPOLE: exocluneasic domaine of POLE
FFPE: formalin‐fixed paraffin embedded
HRM: high resolution melting
MMR‐D: microsatellite deficient
MMR‐P: microsatellite proficient
Mt/Mb: mutations per megabase
NGS: next generation sequencing
TIL: tumor infiltrating lymphocyte
TMB: tumor mutational burden

## 1 Introduction

DNA replication in the S phase of cell cycle involves multiple enzymes including DNA polymerases. These polymerases have activity in both DNA synthesis and DNA repair. Polymerase epsilon, encoded by the *POLE* gene, carries a proofreading (exonuclease) domain allowing error correction during replication ensuring a high‐fidelity replication process. In tumors, *POLE* mutations affecting the exonuclease domain result in a deficient DNA repair activity and a hyper/ultramutated cancer phenotype [1, 2].

It has been recently reported that germline *POLE* mutations are risk factors for colorectal cancer (CRC) and other tumor types, including endometrial cancer (EC) [3]. Somatic *POLE* mutations seem to be more frequent than germline mutations and are found in 5–10% of EC and in 3% of CRC [4, 5, 6]. Three main hotspots have been described in *POLE‐*mutated EC and CRC (codons 286, 411, and 459) and other rare variants, all in the exonucleasic domain, has also been described and associated with a hypermutated phenotype [7]. Interestingly, tumors with mutations in these hotspots are mostly microsatellite stable (MMR‐P). The mean number of genomic mutations in tumors bearing a *POLE* pathogenic mutation appears to be 10‐to 40‐fold higher compared to a population of patients with MMR‐P tumors. As a consequence, *POLE* exonucleasic domain‐mutated tumors define a new hypermutated non MMR‐Deficient (MMR‐D) subtype of cancer.

Hypermutated status is classically assessed by the tumor mutational burden (TMB), an exomic measure of the nonsynonymous mutations per megabase (mt/Mb), and defined by a TMB ≥ 10 mt/Mb. High TMB correlates with increased likelihood for a tumor of harboring immunogenic mutation‐derived neoantigens and benefit derived from immunotherapy in specific tumor types [8]. While TMB has been recently approved in the United States as an agnostic biomarker to indicate immune checkpoint inhibitor [9], TMB assessment is neither available nor approved in most countries because of lack of randomized clinical trials. Recently, *POLE* mutations have been suggested to be an emergent biomarker for response to immunotherapy [10, 11], underlining the need for dedicated screening strategies when TMB is not available in clinical practice.

We previously published a study investigating clinical and molecular profile of *POLE‐*mutated CRC cancers based on available public data [12, 13, 14]. We showed that exonuclease domain *POLE* mutations prevalence was 2.3% in 967 CRC analyzed. The most frequent mutations were P286R/H, V411L (*n* = 3/22), and S459F (*n* = 4/22). The aim of this study was to confirm these observations using exploratory and validation cohorts and to improve *POLE* hypermutated CRC characterization based on clinico‐molecular criteria.

## 2 Materials and methods

### 2.1 Sample selection and design

This study was designed to define clinical, histological, and molecular criteria for the screening of *POLE‐*mutated patients applicable in clinical practice.

First, based on opensource databases (cbioportal.org), we generated an exploratory cohort to define the best clinico‐molecular parameters associated with a *POLE* hotspot mutations.

**Fig. 1:**
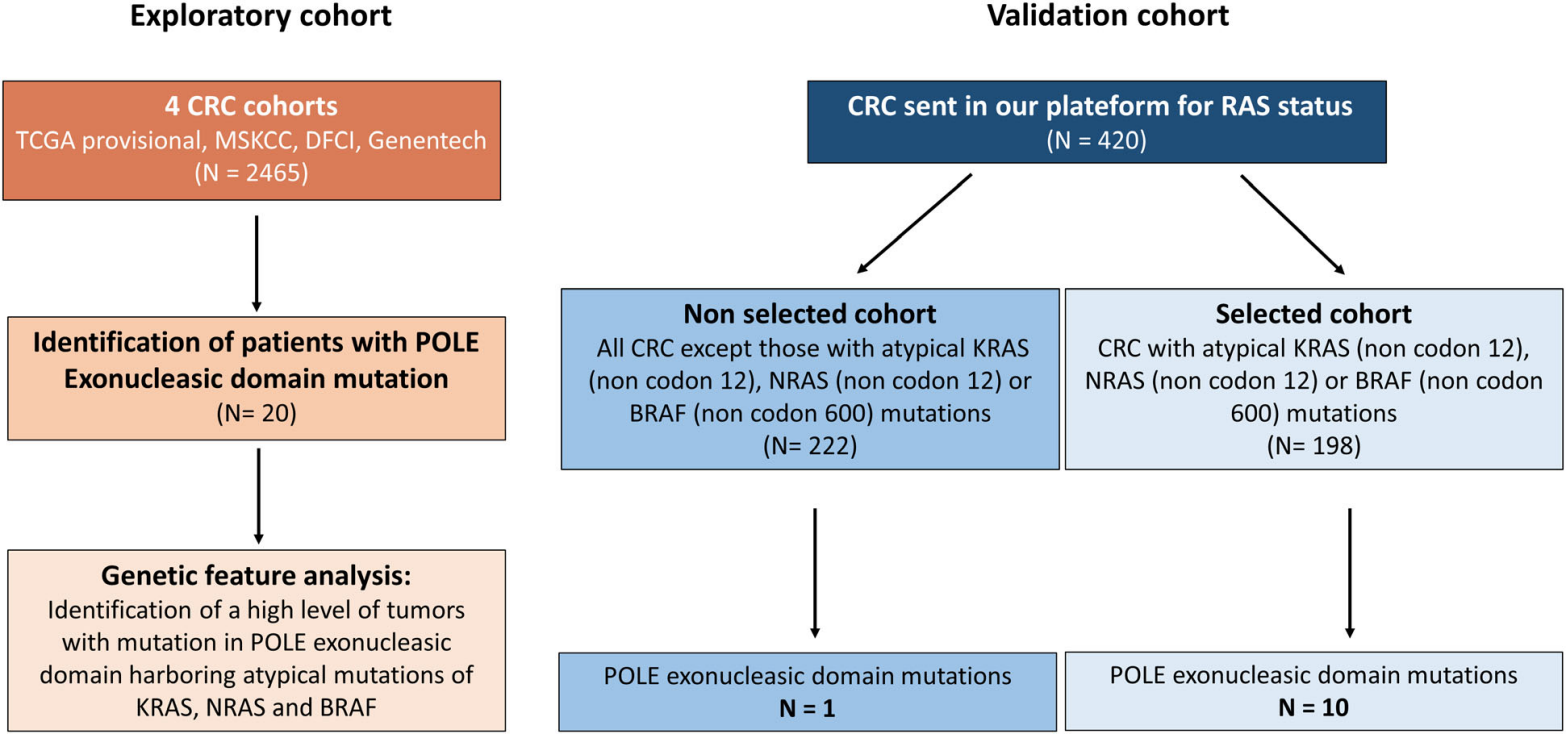
Study design. Exploratory cohort is based on four opensource data: TCGA provisional, MSKCC, DFCI, Genentech (*n* = 2465). Review of available individual genomic data of CRC samples in these four cohorts revealed 20 samples with mutations in the three hotspots described in POLE exonuclease domain. The validation cohort is divided into two groups of CRC: a nonselected group (*n* = 222) and a selected group (*n* = 198) enriched in noncodon 12 mutation in *RAS* and noncodon 600 mutation in *BRAF* genes. POLE status was determined using HRM PCR on both cohorts.

Then, we prospectively sequenced, 420 samples of patients with CRC, corresponding to the validation dataset to evaluate whether the screening method would improve the proportion of hypermutated *POLE‐*mutated MMR‐P patients. Two hundred and twenty‐two samples were allocated to the unselected cohort (unsupervised control screening) while 198 samples were allocated to the selected cohort (experimental screening) defined by the presence of noncodon 12 KRAS mutation, noncodon 600 BRAF mutation, or noncodon 12 NRAS mutation (Fig. 1).

All samples, had previously undergone molecular analysis in our Department of Pathology between 2015 and 2018 for determination of *KRAS* and *NRAS* status, in compliance with French regulations. The prerequisite for sample selection was the availability of residual archival extracted DNA to perform *POLE* PCR analysis. The following items were systematically recorded: age, sex, stage of the disease, location of the tumor, Mismatch Repair (MMR) status, *KRAS*, *NRAS*, *BRAF*, and *PIK3CA* mutational status.

### 2.2 Ethical approval

DNA was extracted from FFPE tissue blocks for medical diagnosis in compliance with French Regulations. The local ethics committee of Henri Mondor University Hospital gave its approval for this study (IRB No. 00011558; 2021–123). Experiements were undertaken with the understanding of each subject. A letter of nonobjection in connection with this study was sent to each patient. The study was performed in accordance with the Declaration of Helsinki.

### 2.3 DNA extraction

All DNA samples had been extracted as previously described [15, 16, 17], after macrodissection when necessary, from formalin‐fixed paraffin‐embedded (FFPE) tissue sections (usually 7 sections, 5‐μm thick) using the Maxwell 16 FFPE Plus LEV DNA Purification Kit IVD (Promega, Charbonnières‐les‐Bains, France), according to the manufacturer's instructions. The DNA was quantified using a Qubit fluorimeter in combination with the Qubit dsDNA HS Array Kit (ThermoFisher Scientific, Waltham, MA, USA).

### 2.4 High resolution melting PCR

*POLE* status was determined using high resolution melting (HRM) performed with a LightCycler® 480 (Roche, Basel, Switzerland) using specific primers (Table S1) for all cases. All samples were tested in duplicate. One positive mutated DNA sample and two wild‐type DNA samples were included as controls in each run. HRM experiments have already been described previously [15]. The final volume of the PCR reaction was 20 μL, containing 10 μL of LightCycler 480 HRM MasterMix (Roche), 3 mm of MgCl_2_, 0.2 μm each primer, 0.5 U of Uracil‐*N*‐Glycosylase, and 20 ng of DNA, as measured by fluorimetry. DNA samples were treated with uracil glycosylase before amplification to avoid artifacts due to cytosine deamination. The cycling protocol was performed as follows: incubation at 37 °C for 10 min, denaturation at 95 °C for 10 min, 45 cycles of amplification (10 s at 95 °C, 15 s at 55 °C, and 30 s at 72 °C), followed by a melting curve (denaturation at 95 °C, hybridization at 40 °C, and melting from 70 to 95 °C). Melting curves from the samples were automatically normalized and analyzed with lightcycler 480 software (Roche).

### 2.5 Next generation sequencing

Patients with mutated profiles identified by HRM PCR were then analyzed using Next Generation Sequencing (NGS) as previously described [15, 16]. For NGS, 10 ng of DNA (as measured by fluorimetry) was amplified using the Ion AmpliSeq™ OST+ V2 panel (ThermoFisher Scientific), which is a multiplex PCR‐based library‐preparation method by which many regions (70–150 bp) that encompass many mutational hotspots including *POLE* codons 286, 411, and 459 are amplified. Amplicons were then digested, barcoded, and amplified by using the Ion Oncomine™ Solid Tumor DNA Kit and Ion Select Barcode Adapter Kit (ThermoFisher Scientific), according to the manufacturer's instructions. After DNA quantification, 25 pm of each library was multiplexed and clonally amplified on ion‐sphere particles (ISP) by emulsion PCR performed on Ion Chef (ThermoFisher Scientific), according to the manufacturer's instructions. The ISP templates were loaded onto an Ion‐520 chip and sequenced on a S5 sequencer with the Ion 510™ & Ion 520™ & Ion 530™ Kit–Chef, according to the manufacturer's instructions. Run performance was assessed and data analyzed with the torrent suite Software v.5.10.0 (ThermoFisher Scientific). Single‐nucleotide variants and small indels were detected using the Variant Caller plug‐in version 5.10.0.18 with low stringency settings (threshold: 2%). The Integrative Genomics Viewer (IGV v 5.01; Broad Institute, Cambridge, MA, USA) was used for visual inspection of the aligned reads.

Tumor mutational burden (TMB) was assessed for patients harboring a *POLE* mutation using FoundationOne® CDX (Roche). F1CDx comprehensive genomic profiling (CGP) has been performed based on the method described by Frampton et al. Tumor mutational burden was evaluated using FoundationOne® method according to Szustakowski et al. [18]. Tumors were considered ultra‐mutated if they contained more than 100 mutations/Megabases (mt/M) hypermutated if they contained between 10 and 100 mt/Mb and with low TMB if they contained less than 10 mt/Mb.

### 2.6 Immunochemistry

IHC analysis was performed in MMR‐P (*n* = 20), MMR‐D (*n* = 20), and *POLE‐*mutated (*n* = 11) tissue sections. IHC was carried out on FFPE tissue sections, as previously described [17], using antibodies against MLH1 (mouse mAb, clone G168.728; Microm Micotech, Brignais, France), PMS2 (mouse mAb, clone A16‐4, 1 : 100; BD Pharmingen, Le Pont de Claix, France), MSH2 (mouse mAb, clone FE11, 1 : 100; Biocare Medical, Pacheco, CA, USA), MSH6 (rabbit mAb, clone EP49, 1 : 100; CliniSciences, Nanterre, France), CD3 (mouse mAb, clone F7.2.38 1 : 50; Agilent Dako, Les Ulis, France), CD8 (mouse mAb, clone C8/144B, 1 : 200; Agilent Dako), CD20 (mouse mAb, clone L26 1 : 500; Agilent Dako), and PDL1 (rabbit mAb, clone QR1, 1 : 100; Diagomics/quartett). IHC was performed on a BOND III or a BOND‐MAX (Leica, Nanterre, France) automated stainer platform. The expression of the four MMR proteins defined a stable phenotype (MMR‐P). Staining pattern consisted in nuclear staining within tumor cells with infiltrating lymphocytes, as positive internal controls. The loss of one or more proteins characterized by a total absence of nuclear staining within tumor cells with a positive labeling of nontumor cells, defined microsatellite unstable phenotype (MMR‐D). PD‐L1 expression was evaluated on tumor cells and immune cells by a pathologist. Membranous cell staining was quantified to give a percentage of positive PD‐L1 cells. Immunolabeling for CD3, CD8, and CD20 were evaluated and quantified using qupath software (version 0.2.0, University of Edinburgh, Edinburgh, UK).

### 2.7 Statistics and public database

The primary endpoint of this study is the prevalence of hotspot *POLE* mutations in the exploratory cohort, and in the validation cohort, control and experimental screening group. The expected prevalence of *POLE* mutation is 0.8% in the unselected – control screening group. We considered that our strategy of screening would be of interest clinically if we reach a prevalence of 5% in the selected‐experimental group. With a power of 0.8 and a bilateral alpha risk of 0.05, we estimated that 424 patients would have to be included in the validation screening cohort to conclude.

Statistical analysis was performed using r software (3.2.2, R Foundation for Statistical Computing, Vienna, Austria). Relationship between qualitative variables were assessed using Chi square tests with Monte Carlo resampling method for multiple hypothesis testing. For IHC, statistical analysis was performed using graphpad prism (San Diego, CA, USA). Mann–Whitney two‐tailed test was used.

## 3 Results

### 3.1 Molecular analysis of POLE‐mutated tumors identified in cBioPortal platform

The first objective of our study was to identify criteria, using an exploratory cohort to screen patients for *POLE* hotspot mutations. Using cBioPortal, four CRC cohorts published with opensource sequencing data were analyzed (Table 1): the TCGA provisional (*n* = 640) [6]; MSKCC (*n* = 1134) [19]; DFCI (*n* = 619) [20]; and Genentech (*n* = 72) [21]. Review of available individual genomic data of CRC samples in these four cohorts revealed 20 samples (0.8%) with mutations in the three hotspots described in *POLE* exonuclease domain (codons 86–460). The mean age at diagnosis was 59 (Table 1). Patients with *POLE* mutation were more frequently men (65% vs 25% female and 10% unknown) and tumors were mostly located in right colon (50%). As expected, most of *POLE* mutations affected codon 286 (P286R/H; 45%), codon 459 (S459F; 30%), and codon 411 (V411L; 25%). Interestingly, these *POLE* mutations were predominantly associated with *KRAS*, *NRAS*, *BRAF*, and/or *PIK3CA* mutations, commonly found in colorectal cancers. But unlike classical CRC, *POLE‐*mutated tumors were strongly associated with unusual mutations, which exhibit rare prevalence in these genes: 65% harbored noncodon 12 *KRAS* mutations (G13D; V14I; D57N; E98*; K117N; A146T; K147T/E or K170Q); 20% harbored noncodon 12 *NRAS* mutation (Q61R; E132K; R167*); 40% harbored a non‐V600E *BRAF* mutation (F294L; Y633C; L312P; S602Y; Q356K; R354*; S102Y; L567V; R389C; F247L); 55% harbored a *PIK3CA* mutation. In these series, 55% of the cases presented multiple concomitant unusual mutations. Regarding MMR status, 65% were MMR‐P, 5% were MMR‐D, and 30% were unknown. Altogether, these results suggest that hotspot *POLE‐*mutated tumors in CRC have molecular characteristics, such as MMR‐P status and noncodon 12 mutations of *KRAS/NRAS* and noncodon 600 *BRAF* mutation (*P* = 0.02).

**Table 1:** Characteristics of *POLE‐*mutated patients in opensource data.

*POLE‐*mutated cases (%):	20 (0.8)
Mean of age (year) [Range]	59 [24–86]
Location (%)	
Right colon	10 (50)
Left colon	4 (20)
Rectum	4 (20)
Unknown	2 (10)
Sex (%)	
M	13 (65)
F	5 (25)
Unknown	2 (10)
*POLE* mutation (%)	
Codon 286	9 (45)
Codon 459	6 (30)
Codon 411	5 (25)
*KRAS* mutation (%)	
WT	7 (35)
Codon 12 mutation	0 (0)
Noncodon 12 mutation	13 (65)
*NRAS* mutation (%)	
WT	16 (80)
Codon 12 mutation	0 (0)
Noncodon 12 mutation	4 (20)
*BRAF* mutation (%)	
WT	12 (60)
Codon 600 mutation	0 (0)
Noncodon 600 mutation	8 (40)
*PIK3CA* mutation (%)	
WT	9 (45)
Mutation	11 (55)
MSI status (%)	
MMR‐P	13 (65)
MMR‐D	1 (5)
Unknown	6 (30)

### 3.2 Screening for POLE‐mutated CRC in the exonuclease domain in a cohort of nonselected or cases selected for unusual mutation of KRAS, NRAS, and BRAF

Based on these findings, we prospectively assessed if selecting cases with noncodon 12 *KRAS/NRAS* mutations and noncodon 600 *BRAF* mutation would increase the prevalence of *POLE‐*mutated MMR‐P CRC in the exonuclease domain compared with an unselected population.

Between June 2013 and September 2018, a total of 420 patients, screened for RAS status in our lab, were included in this study and divided into two groups: a nonselected control cohort (*n* = 222) of patients and a selected experimental cohort (*n* = 198) specifically selected on the presence of noncodon 12 *KRAS/NRAS* mutations and noncodon 600 *BRAF* mutation associated with a MMR‐P status. Patient characteristics of these two groups are shown in Table 2. The mean age of these patients was 68 for both cohorts, ranging from 27 to 96 for the nonselected cohort and from 27 to 94 in the selected cohort. Male to female sex ratio was 1.3 for both cohorts. All stages CRC were included in the study. At the molecular level, tumors were predominantly MMR‐P in both cohort (91% and 95% for the nonselected and selected cohort, respectively). The nonselected cohort contained mostly cases with a *KRAS/NRAS/BRAF/PIK3CA* wild‐type status or with typical mutations of these genes (codon 12 mutation for *KRAS*, codon 12 mutation for *NRAS* and codon 600 mutation for *BRAF*). As defined, the selected cohort had a high rate of tumors with noncodon 12 *KRAS* mutation (87% vs 15% in nonselected cohort) and noncodon 600 *BRAF* mutation (15% vs 4% in the nonselected cohort).

**Table 2:** Clinical and molecular characteristics of patients included in the two cohorts.

	Nonselected cohort	Selected cohort
*n*	222	198
Age (years)	68 (27–96)	68 (27–94)
Sex		
Male	126 (57%)	112 (57%)
Female	96 (43%)	86 (43%)
Stage		
I	5 (2%)	4 (2%)
II	45 (20%)	29 (15%)
III	69 (31%)	74 (37%)
IV	42 (19%)	39 (20%)
Unknown	61 (28%)	52 (26%)
Location		
Right	77 (35%)	69 (35%)
Left	76 (34%)	57 (29%)
Rectum	48 (22%)	46 (23%)
Unknown	21 (9%)	26 (13%)
MMR status		
MMR‐P	202 (91%)	188 (95%)
MMR‐D	10 (4.5%)	0 (0%)
Unknown	10 (4.5%)	10 (5%)
*KRAS* status		
Wild‐type	120 (54%)	26 (13%)
Typical mutation (codon 12)	63 (28%)	0 (0%)
Atypical mutation	33 (15%)	172 (87%)
Unknown	6 (3%)	0 (0%)
*BRAF* status		
Wild‐type	122 (55%)	155 (78%)
Typical mutation (codon 600)	19 (9%)	0 (0%)
Atypical mutation	10 (4%)	29 (15%)
Unknown	71 (32%)	14 (7%)
*NRAS* status		
Wild‐type	139 (63%)	175 (88%)
Mutation	13 (6%)	8 (4%)
Unknown	70 (31%)	15 (7.5%)
*PIK3CA* status		
Wild‐type	126 (57%)	155 (78%)
Mutation	23 (10%)	29 (15%)
Unknown	73 (33%)	14 (7%)
*POLE* status		
Wild‐type	221 (99.5%)	188 (95%)
Mutation (exonuclease domain)	1 (0.5%)	10 (5%)

We identified 10 tumors out of 198 (5%) carrying *POLE* mutation in the exonuclease domain in the selected cohort whereas only one tumor out of 222 (0.5%) was mutated in the nonselected cohort (Chi‐square test, *P* = 0.0032). Of note, the latter mutated sample was observed in a MMR‐D tumor while the 10 other mutated tumors were all MMR‐P. These results highlight the benefits of screening preferentially CRC samples with *KRAS/BRAF/NRAS* unusual mutation(s) to improve the identification of *POLE‐*mutated patients in the exonuclease domain. The molecular screening criteria lead to an enrichment by 10‐fold in the prevalence of *POLE* mutations.

### 3.3 Molecular characteristics of the POLE‐mutated tumors identified during the study

The characteristics of the 11 cases of *POLE‐*mutated tumors in the exonuclease domain are presented in Table 3. Four *POLE* mutations were found on codon 286, two on codon 411, one on codon 425, one on codon 459, one on codon 461, and two on codon 464. One of these mutations on codon 464 was a silent mutation (V464V, case number 11) and corresponds to the MMR‐D case identified in the nonselected cohort. The median age of these patients was 54.2, ranging from 31 to 73 years old. They were predominantly male (*n* = 9, 82%) and CRC were mostly left‐sided (*n* = 6, 55%). Except case number 11, all tumors were MMR‐P.

**Table 3:** molecular characteristics of POLE‐mutated patient.

	*POLE‐*mutated patients
n	11
Age (years)	54.2 (31–73)
Sex	
Male	9 (82%)
Female	2 (18%)
Location	
Right	3 (27%)
Left	6 (55%)
Rectum	1 (9%)
Unknown	1 (9%)
MMR status	
MMR‐P	10 (91%)
MMR‐D	1 (9%)
KRAS status	
Wild‐type	2 (18%)
Typical mutation (codon 12)	0 (0%)
Atypical mutation	9 (82%)
BRAF status	
Wild‐type	9 (82%)
Typical mutation (codon 600)	0 (0%)
Atypical mutation	2 (18%)
NRAS status	
Wild‐type	10 (91%)
Mutation	1 (9%)
PIK3CA status	
Wild type	10 (91%)
Mutation	1 (9%)

Among these 11 tumors, 9 (82%) carried a noncodon 12 *KRAS* mutation (G13D; A59T; N116H; K117N; A146V/T), associated or not with *BRAF* or *PIK3CA* mutations. Two tumors (18%) carried a noncodon 600 *BRAF* mutation (D454V or D594G). One tumor (9%) carried a double *NRAS* mutation (Q61R and T58A) and one tumor (9%) carried a *PIK3CA* mutation (E542K). In this series, *POLE* mutation was associated with *KRAS* mutation on codon 146. The mutational burden score of theses tumors was assessed with FoundationOne® CDX (Roche). Among the 11 POLE‐mutated cases, 6 were ultramutated ≥ 100 mutations/Megabases (mt/Mb, cases number 1, 2, 5, 6, 7, 8) 3 were hypermutated ≥ 10 and < 100 mt/Mb (cases number 9, 10, 11) and 2 had low TMB < 10 mt/Mb (cases number 3 and 4).

### 3.4 Evaluation of the infiltration of POLE‐mutated tumors by immune cells

**Fig. 2:**
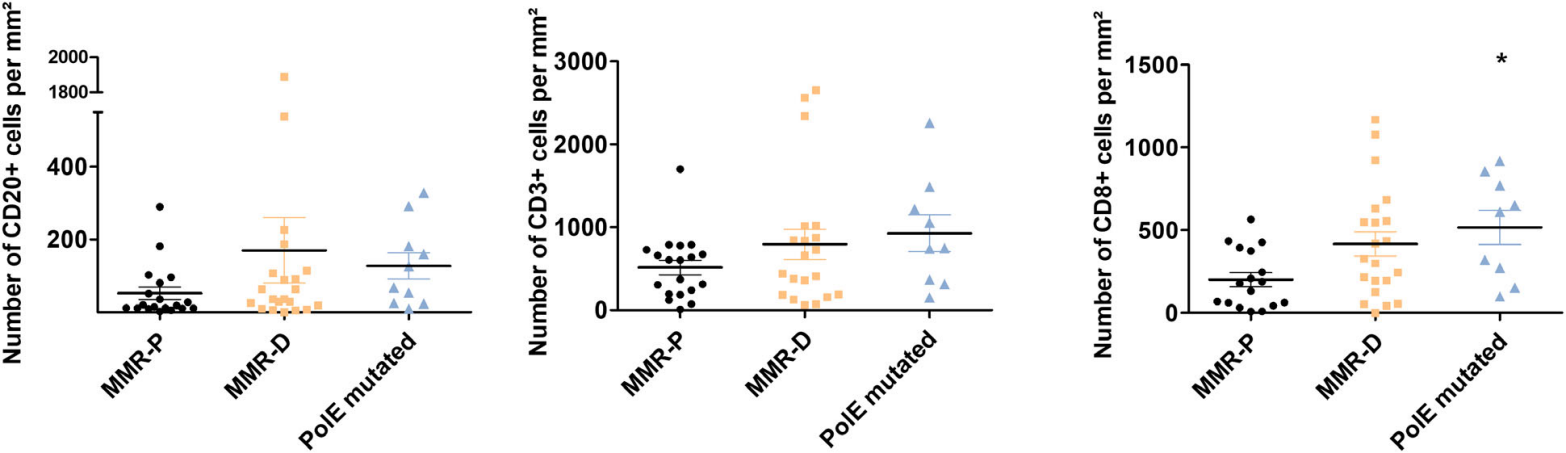
Analysis of CD3, CD8, and CD20 immunolabeling in MMR‐P, MMR‐D, and *POLE‐*mutated CRC. CD3, CD8, and CD20 immunolabelings were performed in MMR‐P (Mismatch Repair‐proficient, *n* = 20), represented in black circles, MMR‐D (Mismatch Repair‐deficient, *n* = 20), represented in orange squares, and *POLE* (*n* = 10) mutated CRC (colorectal carcinomas), represented in blue triangles, to compare the level of the different tumor infiltrated lymphocytes (TILs). Data are represented as mean ± SEM (standard error of the mean). Black bars represent mean. Statistical analysis were performed using Mann–Whitney two‐tailed test (nonsignificant values are not indicated, * *P* < 0.05).

**Fig. 3:**
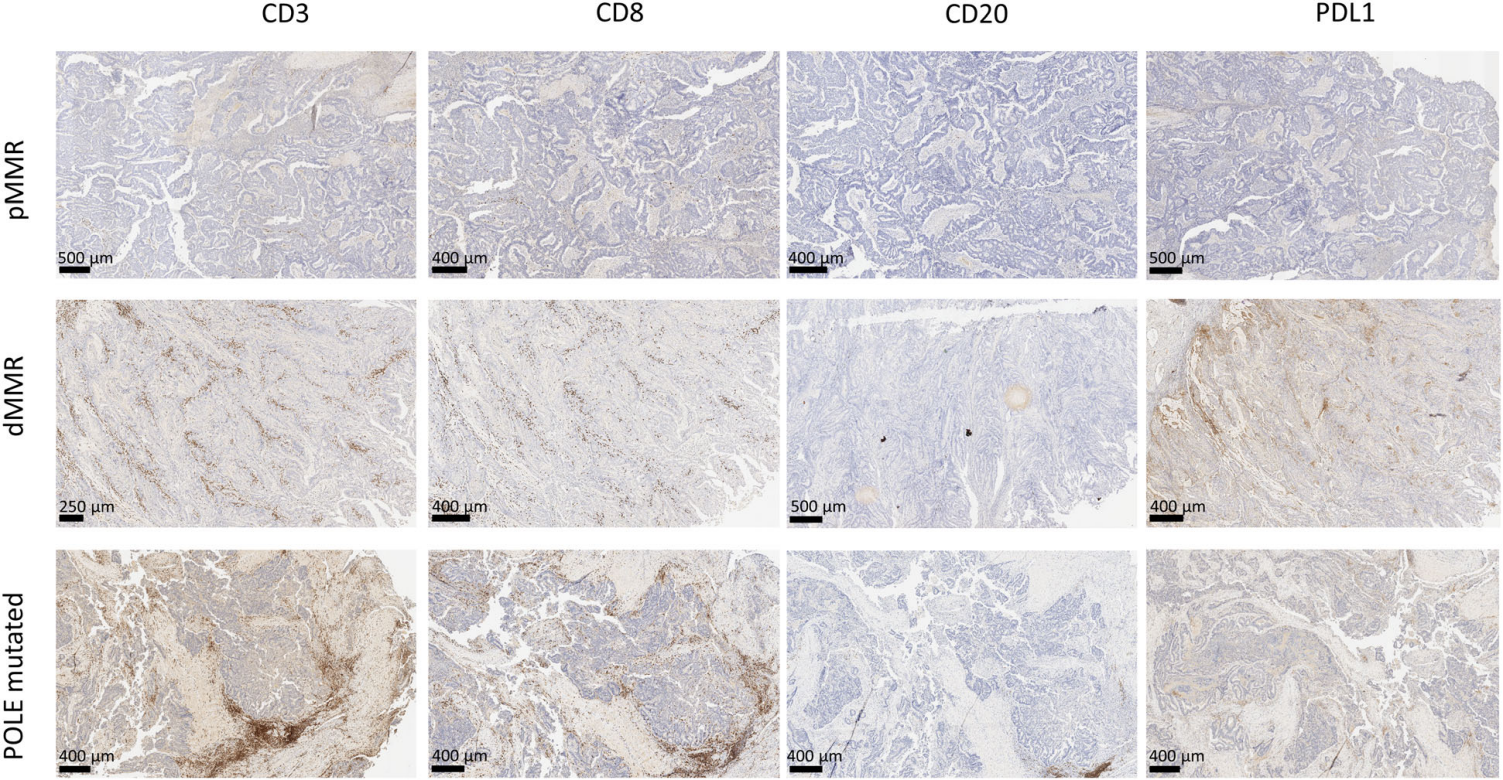
Representative CD3, CD8, CD20, and PDL1 immunolabeling in MMR‐P, MMR‐D, and *POLE‐*mutated CRC tissue sections. CD3, CD8, CD20, and PDL1 immunolabelings for MMR‐P (Mismatch Repair‐proficient, *n* = 1), MMR‐D (Mismatch Repair‐deficient, *n* = 1) and *POLE‐*mutated (*n* = 1) CRC (colorectal carcinomas) were represented to illustrate data shown in Fig. 2. MMR‐P tumors display low TIL levels while MMR‐D and *POLE*‐mutated tumors display higher levels. In *POLE‐*mutated tumors, PD‐L1 is frequently negative or with low staining. Scale ranging from 250 to 500 μm.

Several studies have shown that MMR‐D CRC have higher rate of CD8^+^ tumor‐infiltrating lymphocytes (TILs) than MMR‐P tumors [22, 23, 24]. We therefore assessed if *POLE‐*mutated CRC tumors had an immune profile closer to MMR‐D than other MMR‐P tumors. We performed IHC to compare the density of CD3^+^, CD8^+^, and CD20^+^ TILs, between MMR‐P (*n* = 20), MMR‐D (*n* = 20), and *POLE‐*mutated (*n* = 11) ‐CRC. Our results show that MMR‐P tumors have the lowest densities of CD3^+^ CD8^+^ and CD20^+^ TILs as compared to MMR‐D and *POLE‐*mutated tumors (Fig. 2). No significant difference was observed between the three groups for CD3^+^ TILs. However, CD8^+^ TILs were significantly higher in *POLE‐*mutated CRC (*P* = 0.0073) and in MMR‐D tumors (*P* = 0.0493) than in MMR‐P tumors. Similarly, CD20^+^ TILs were higher in *POLE‐*mutated tumors than in MMR‐P tumors (*P* = 0.0310; Fig. 2). Representative immunostaining for MMR‐P, MMR‐D, and *POLE*‐mutated CRC are illustrated in Fig. 3. Immunohistochemistry staining was then performed to evaluate PD‐L1 expression in *POLE‐*mutated tumors. Results showed that tumor cells were most frequently PD‐L1 negative (*n* = 8) or with low staining (tumor cells less than 10%, *n* = 2; Table 4) suggesting that *POLE‐*mutated tumors are not associated with PD‐L1 expression. Representative PD‐L1 staining is shown in Fig. 3.

**Table 4:** Clinicopathological characteristics of ed*POLE‐*mutated patients.

Sex	Age	Localization	Stage	MMR status	Mutations	PolE mutation	Mutational burden score	IHC PDL1 tumor cells	IHC PDL1 immune cells
M	47	Rectum	NP	MMR‐P	KRAS N116H	p.(Ser461Thr)	226.95	<1%	40%
M	63	Left	T4bN0	MMR‐P	KRAS A59T; BRAF D454V	p.(Pro286Arg)	189.13	0%	0%
F	73	Sigmoid	NP	MMR‐P	BRAF D594G	p.(Val464Ala)	3.78	0%	10%
M	64	Unknown	M1 (liver)	MMR‐P	NRAS Q61R T58A	p.(Lys425Arg)	3.78	0%	10%
M	31	Sigmoid	NP	MMR‐P	KRAS K117N	p.(Val411Leu)	417.34	20%	5%
M	55	Left	NP	MMR‐P	KRAS A146T; PIK3CA E542K	p.(Pro286Arg)	116	0%	0%
F	65	Right	NP	MMR‐P	KRAS A146V	p.(Val411Leu)	142.48	0%	0%
M	36	Left	T4bN0M1	MMR‐P	KRAS A146T	p.(Pro286Arg)	218.13	5%	5%
M	42	Right	NP	MMR‐P	KRAS A146T	p.(Pro286Arg)	78.17	10%	10%
M	61	Sigmoid	NP	MMR‐P	KRAS G13D	p(Ser459Phe)	49.17	0%	60%
M	59	Right	T4aN2b	MMR‐D	KRAS G13D	p.(Val464Val)	56.74	<1%	0%

Altogether, these data indicate that *POLE‐*mutated CRC had an immune profile closer to MMR‐D than MMR‐P tumors, suggesting they could exert an antitumor activity in *POLE‐*mutated CRC so as in MMR‐D CRC, independently of PD‐L1 expression.

## 4 Discussion

Exonucleasic domain *POLE* mutations occur in 1–2% of MMR‐P CRC responsible for hypermutated phenotype and are emergent predictive biomarkers for response to immune checkpoint inhibitors. To our knowledge, this is the first study which demonstrate that ed*POLE‐*mutated tumors are associated with high prevalence of noncodon 12 *KRAS/NRAS* mutations and noncodon 600 *BRAF* mutation. Furthermore, our preliminary data show that this observation is not found in other types of cancer and seems specific to CRC (data not shown). Restricting the ed*POLE* mutation screening to patients with tumors harboring these unusual mutations in *KRAS*, *NRAS*, and/or *BRAF* genes lead to an enrichment of the prevalence of ed*POLE* mutations up to 5% while in an unselected population the prevalence was only 0.5%. Clinically, ed*POLE* tumors were mostly observed in men with left‐sided CRC, with a noncodon 12 *KRAS* mutation and hyper/ultramutated MMR‐P. Microscopically, ed*POLE‐*mutated tumors displayed high CD8^+^ TILs infiltration and were not associated with high expression of PDL‐1 confirming previous observations [25, 26]. Interestingly, a study performed by Domingo et al. [26] on 6517 colorectal cancers, showed 66 *POLE‐*mutated tumors. In this cohort, only one case of *POLE‐*mutated tumors was associated with *KRAS* mutation. Nevertheless, only *KRAS* exon 2 (codons 12 and 13) and *BRAF* codon 600 were analyzed in this study. Clinical observations are consistent with ours: *POLE‐*mutated tumors were observed in young men but predominantly right‐sided, whereas only 3 out of 11 *POLE‐*mutated tumors were right‐sided in our study. Another study performed on stage II CRC showed a higher percentage of *edPOLE‐*mutated patients (3.1%) than in the four CRC cohorts published (Table 1) and clinicopathological characteristics are also identical to those mentioned above [27].

In our study, the method used to detect these mutations is based on a qualitative, rapid, and low‐cost PCR. Identified *POLE‐*mutated tumors need to be further sequenced to determine the exact mutation and its pathogenicity. As TMB is not an approved biomarker worldwide and because its assessment requires large sequencing panels or whole exome sequencing, *POLE‐*targeted sequencing seems a seducing alternative when TMB is not available. Our study shows that the presence of a noncodon 12 *KRAS* or *NRAS* mutation should lead clinicians and biologists to look for the presence of *POLE* mutation. Moreover, recent reports also suggest that the benefit derived from immunotherapy in high TMB CRCs is limited to patients with tumors displaying DNA repair impairment such as in MMR‐D and POLE proofreading deficiency. These data highlight the need to assess the underlying cause of high TMB in CRC to offer immunotherapy, making *POLE* assessment necessary for MMR‐P tumors. However, our study shows that *POLE* exonucleasic domain mutations are not always associated with a high TMB. In our series, one patient was MMR‐D, with a silent ed*POLE* mutation V464V. ClinVar predicts this variant as likely benign and might have low impact on Polymerase Epsilon function. In this case, high TMB was probably due to MMR‐D phenotype instead of ed*POLE* mutation. Further studies are thus needed to assess which nonhotspot *POLE* mutations are pathogenic and driver in order to identify those who are correlated with a good response to immunotherapy.

First results of the program AcSé Nivolumab including 16 patients with MMR‐P *POLE‐*mutated tumors showed an overall response rate of 50% in patients tumors harboring pathogenic exonucleasic domain mutations, all in advanced CRC. Conversely, no response was observed for the patients carrying nonpathogenic mutations. Comparing patients harboring nonpathogenic and pathogenic/unknown significance variants, a survival benefit was also observed (mOS = 5.3 months vs not reached, *P*‐value = 0.003) [28]. Furthermore, several case reports describe immune checkpoint inhibitor efficiency in *POLE‐*mutated CRC patients, regardless of the expression of PDL‐1 [29, 30]. As patients with ed*POLE*‐mutated tumors seems to be extreme responders to immune checkpoint blockade, early identification of these patients in localized or advanced setting could allow clinicians to offer early immunotherapy‐based strategies to avoid chemo/radiotherapy and improve clinical outcomes.

Finally, this study raises questions about the role of unusual mutations in the carcinogenesis of ed*POLE‐*mutated tumors. Poulin et al. [31] demonstrated that there are different biochemical properties of *KRAS* mutations. *KRAS* G12D would activate the MAPK pathway more strongly than the *KRAS* A146T. In addition, global proteomic analysis revealed that *KRAS* A146T clustered more closely with *KRAS* WT than with *KRAS* G12D. Other data reinforce the hypothesis that not all *KRAS* mutations are equivalent. For instance, patients with *KRAS* G13D mutation would have a better outcome compared to patients mutated on codon 12 after cetuximab treatment [32]. Nevertheless, these data remain controversial and no clinical trial has demonstrated cetuximab benefit for patients with *KRAS* G13D [33, 34, 35].

In this context, in contrast to typical *KRAS‐*mutated tumors, the initiating event of carcinogenesis could be the *POLE* mutation, as it has been shown by Temko et al. [36]. By occurring early, replication errors are no longer repaired and accumulate in the cells, resulting in a large number of neoantigens and a high TMB.

## 5 Conclusion

To conclude, this study improves our molecular understanding of *POLE‐*mutated tumors and should encourage the search for *POLE* mutations in tumors with an atypical *KRAS* mutation, especially in young men with an MSS phenotype. Nevertheless, more work is needed to understand why high prevalence of unusual mutations in the *RAS* and *BRAF* genes are observed in these patients.

## Conflict of interest

The authors declare no conflict of interest.

## Author contribution

LF, JCo, AP, RB, and LL performed experiments. LF, JCo, AD, and C‐TN analyzed immunolabeling. AP, BR, and CT designed the study. AD and MO participated in the study conception. LF wrote draft manuscript. LF, JCo, JCa, RB, LL, MO, and AP participated in sample selection. AD, JCa, EL, IS, CT, BR, and AP revised draft manuscript. All authors read and approved the final manuscript.

### Peer Review

The peer review history for this article is available at https://publons.com/publon/10.1002/1878‐0261.13257.

## Supporting information


**Table S1.** Primers used for *POLE* HRM PCR.

## Data Availability

All data generated or analyzed during this study are included in this published article.
