# Supplementary material for: High prevalence of unusual KRAS, NRAS, and BRAF mutations in POLE ‐ hypermutated colorectal cancers
Source: Mol Oncol. 2022 Jul 14;16(17):3055–65. doi: 10.1002/1878-0261.13257 (PMC9441000; doi:10.1002/1878-0261.13257)
Supplement: Supplementary file 1 — Table S1. Primers used for POLE HRM PCR. [file MOL2-16-3055-s001.docx]

**Supporting Information**

**Supplementary Table 1. Primers used for *POLE* HRM PCR**

|  | **Primers (5’→ 3’)** | |
| --- | --- | --- |
| **Exon 9** | L 1 | ATTGAGACGACCAAACTGCC |
|  | R1 | CCCATCCCAGGAGCTTACTT |
| **Exon 13** | L2 | GGGCTGCATGTTAGAATCATCT |
|  | R2 | AGCTCCACGGGATCATAGC |
| **Exon 14** | L1 | CTCTCTGGCGTTCTCTCCTC |
|  | R1 | CAGATAATGCTCACCTCGTCG |
